# Opportunistic Salpingectomy in Non‐Gynecologic Surgeries: Barriers and Facilitators From a Healthcare Provider Perspective

**DOI:** 10.1002/cam4.70945

**Published:** 2025-05-09

**Authors:** Charlotte Fisch, Tamar Gootzen, Joanne de Hullu, Philip de Reuver, Diederik Somford, Simon Nienhuijs, Jurgen Piek, Rosella Hermens

**Affiliations:** ^1^ Department of Obstetrics and Gynecology Radboud University Medical Centre Nijmegen the Netherlands; ^2^ Department of Surgery Radboud University Medical Centre Nijmegen the Netherlands; ^3^ Department of Urology Canisius‐Wilhelmina Hospital Nijmegen the Netherlands; ^4^ Department of Surgery Catharina Hospital Eindhoven the Netherlands; ^5^ Department of Obstetrics and Gynecology, and Catharina Cancer Institute Catharina Hospital Eindhoven the Netherlands; ^6^ Department of IQ Healthcare Radboud University Medical Centre Nijmegen the Netherlands

**Keywords:** fallopian tube, implementation, opportunistic salpingectomy, ovarian carcinoma

## Abstract

**Objective:**

This study identifies barriers and facilitators for implementing opportunistic salpingectomy (OS) during non‐gynecological abdominal surgeries from a healthcare provider perspective.

**Methods:**

From October 2023 to July 2024, a mixed‐method study was conducted. The qualitative phase involved semi‐structured focus group interviews and individual interviews with specialists in surgery (gynecologists, general surgeons, urologists, and residents) and policymakers to identify barriers and facilitators for implementing OS during non‐gynecological surgery. The quantitative phase consisted of a cross‐sectional web‐based survey assessing the importance of these barriers and facilitators. The study utilized the standardized implementation frameworks to categorize the factors into six domains: innovation, patient, healthcare professional, social setting, organization, and economic and financial context.

**Results:**

In the qualitative phase, 38 healthcare professionals and policymakers identified 38 barriers and 28 facilitators. Barriers were found in all domains and mainly included increased workload, unclear invoicing, and variations in eligible surgeries. Facilitators included the poor prognosis of ovarian cancer, simplicity of OS, and availability of counseling materials. The quantitative survey revealed that 75% of gynecologists, 60% of surgeons, and 61% of urologists supported offering OS during non‐gynecological abdominal surgeries. Barriers identified included the ambiguity regarding which patients are eligible for OS, the perceived complication risks of OS, the increased workload as a result of adding OS, and the unclarity around invoicing an OS. Facilitators included the poor prognosis of ovarian cancer, the availability of uniform counseling materials, education on counseling and technical performance of OS, involvement of a gynecologist during the counseling, and clear agreements between the departments within hospitals.

**Conclusions:**

Key barriers to OS implementation in non‐gynecological surgeries include unclear invoicing and increased workload, while significant facilitators are the availability of counseling materials and education on counseling and technical performance of OS. Addressing these barriers and leveraging facilitators could enhance OS adoption, potentially reducing ovarian cancer incidence.

## Graphical abstract

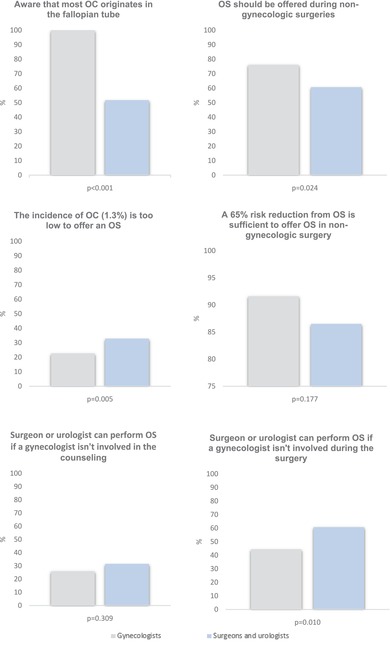

## 1 Introduction

Preventing OC is crucial, given its status as the most lethal type of gynecological cancer [1]. A woman's lifetime risk of developing OC is approximately 1 in 87 [2]. In general, OC is treated aggressively with a combination of debulking surgery and chemotherapy. Despite the aggressive treatment, the majority of patients will experience a recurrence and develop resistance to chemotherapy, contributing to a poor 5‐year survival rate of approximately 29% [1, 3]. Roughly 80% of OCs are diagnosed at an advanced stage due to delayed identification, caused by a late onset of unspecific symptoms [1, 3]. Despite extensive research, screening methods (with ultrasound and/or Cancer Antigen 125 (CA 125)) have proven to be ineffective [4]. Given the absence of effective screening programs and the limited treatment options, focus should shift towards primary prevention of OC.

Roughly 20 years ago, an important discovery was made, showing that the fallopian tube, rather than the ovary itself, serves as the main origin of most OCs [5, 6, 7]. As a result, removal of the Fallopian tubes significantly reduces OC incidence [8, 9, 10]. The relatively low lifetime ovarian cancer risk of approximately 1.3% does not justify preventive surgery alone, due to risks linked to anesthesia and surgery, which do not outweigh those of OC [2]. However, when abdominal surgery is already being performed for another reason, there is an opportunity for the prevention of OC by removing the fallopian tubes simultaneously, while leaving the ovaries in situ to preserve hormone production [11]; this is the so‐called opportunistic salpingectomy (OS). Several international gynecologic societies advocate discussing OS with women undergoing abdominal surgery after completion of childbearing [12]. Among gynecologists, consultations about OS are already common practice in many countries, in which the majority of women undergoing gynecological surgery are informed about the opportunity and choose to undergo OS.

More than half of women in developed countries will undergo intra‐abdominal surgery during their lifetime, presenting a significant opportunity for the prevention of ovarian cancer (OC) [13]. One clinical study specifically investigated the feasibility of performing OS during non‐gynecological intra‐abdominal surgery, particularly during cholecystectomy, and found substantial patient acceptance along with high success rates for OS [14]. Furthermore, economic evaluations have shown that OS not only reduces the incidence of OC but also decreases healthcare costs [15, 16]. Since these findings, multiple professionals have advocated implementing OS during non‐gynecological intra‐abdominal surgeries [17, 18].

Nonetheless, integrating OS into non‐gynecologic practice is challenging, especially since this integration requires adaptation by both healthcare professionals and policy agreements [19]. A crucial step towards future implementation is the development of a tailored implementation strategy based on barriers and facilitators found among different stakeholders [20, 21]. Therefore, this study aims to identify barriers and facilitators of implementing OS in non‐gynecological surgery from professionals' and policymakers' perspectives.

## 2 Methods

### 2.1 Study Design

**FIGURE 1:**
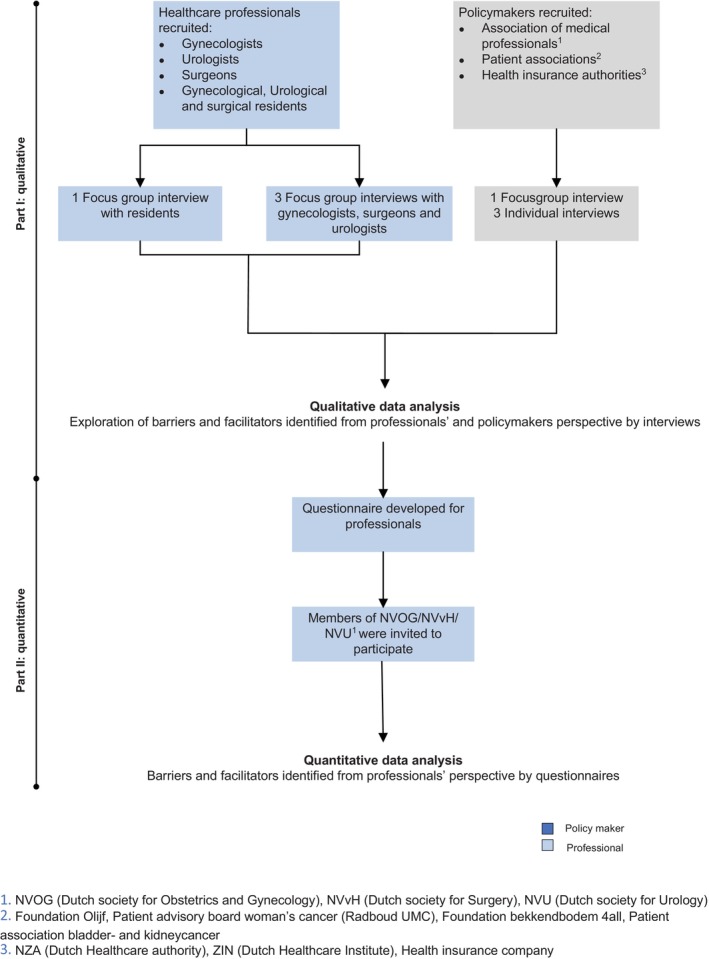
Flowchart of the study design.

We conducted a mixed‐method study between October 2023 and July 2024. In the qualitative phase, we held semi‐structured focus groups and individual interviews with healthcare professionals and policymakers to identify barriers and facilitators in implementing OS during non‐gynecologic surgery. Subsequently, in the quantitative phase, the importance of the identified barriers and facilitators among professionals was assessed in a cross‐sectional web‐based survey (Figure 1). During both phases, barriers and facilitators were categorized with the implementation frameworks of Flottorp et al. [21] and Grol and Wensing [20] into the following domains: innovation (characteristics of the intervention itself), patient (factors related to patients' needs, preferences, knowledge, attitudes or behaviors), healthcare professional (factors affecting professionals' knowledge, attitudes, motivation or skills), social setting (influence of colleagues, teams, or culture), organization (resources, management, and workflows within healthcare organizations), and economic and financial context (financial incentives, costs, or funding availability).

The study was carried out according to the Consolidated criteria for Reporting Qualitative studies (COREQ) (Supporting Informations S1) and the Checklist for Reporting Results of Internet E‐Surveys (CHERRIES) (Supporting Informations Data S2) [22, 23].

This study was not subjected to the Dutch ‘Medical Research Involving Human Subjects Act’, as assessed by the institutional review board of the Radboud University Medical Center (reference no. 2023/16486).

The research team consisted of seven members with previous expertise in implementation research: C.F. (MD, PhD candidate), T.G. (MD, PhD candidate), P.R. (MD, PhD, gastrointestinal and oncologic surgeon), R.S. (MD, PhD, urologist), J.P. (MD, PhD, gyne‐oncologist), J.H. (MD, PhD, gyne‐oncologist, associate professor gynecologic oncology), R.H. (PhD, professor in personalized oncology care).

### 2.2 Qualitative Phase

#### 2.2.1 Healthcare Professionals

A panel of gynecologists, general surgeons, urologists, and residents from these three specialties was recruited through targeted invitations by e‐mail by one researcher (C.F.). We aimed to create a diverse group by recruiting professionals from different regions in the Netherlands, from university‐affiliated and non‐university hospitals and with different levels of work experience. Recruited professionals were invited to propose other professionals eligible to join the study. Participants were included if they were licensed professionals or residents in training. Before the focus group interview, professionals completed a short web‐based survey (electronic data management system CastorEDC) regarding their professional experience (e.g., years of experience, clinical setting, number of operations, knowledge on OS and experience with OS). The survey included informed consent for audio recording of the sessions. In every focus group, 8–12 participants were planned to participate [24, 25].

#### 2.2.2 Policymakers

To discuss the potential clinical and financial implications of OS during non‐gynecological surgery, policymakers in the fields of medical professionals, patient advocacy, and care financing were asked to participate in a focus group or individual interviews. For the focus group, policymakers of medical professionals' associations—NVOG (Dutch Society for Obstetrics and Gynecology), NVvH (Dutch Society for Surgery), NVU (Dutch Society for Urology)—as well as patient associations (Radboudumc Patient Advisory Board for woman's cancer, Foundation Olijf, Foundation bekkenbodem 4all, patient association bladder‐and kidney cancer) were purposively e‐mailed by one researcher (C.F.). Policymakers of Health insurance authorities (insurance advisors, insurance companies), the National Healthcare Institute (ZIN) and Dutch Healthcare Authority (NZA) were e‐mailed for participation in individual interviews. Informed consent for audio recording was obtained verbally during the interview sessions.

#### 2.2.3 Data Collection

Separate semi‐structured interview guides were developed beforehand by two researchers (C.F., medical doctor and PhD candidate) and R.H. (professor in implementation research), drawing upon literature and research experience but also allowing interaction between participants. We included open‐ended questions supplemented by optional questions. Healthcare professionals were inquired about their knowledge of OC and OS, and their opinions on implementing OS during non‐gynecologic surgeries. The interview guide for policymakers focused on implementing OS and its consequences on OC in the general population, the organization of care, and healthcare costs/savings. Two gynecologists (JP, JdH) assessed and approved the interview guides. The focus groups and interviews were performed online (videoconference platform Microsoft Teams) between October 2023 and March 2024. In each focus group session, at least three research team members were present: one as moderator, one as notekeeper, and one providing technical support. The moderator (C.F.) is experienced in conducting qualitative research. During the three individual interview sessions, at least two research team members were present: the moderator and the notekeeper. All focus groups and interviews were audio recorded after the moderator introduced herself and the research team, explained the research question, presented background information on OS, the interview procedure, and funding. Then participants introduced themselves and explained their backgrounds. Participants were given the opportunity to conclude remarks at the end. For privacy reasons, identifiable data was not audiotaped. Field notes were obtained. Data were regularly examined throughout the collection process, and saturation was considered achieved when the information became repetitive, with no new themes or perspectives emerging during the focus groups or interviews.

#### 2.2.4 Data Analysis

All interviews were transcribed verbatim. Transcripts were not returned to participants for feedback. Two researchers (C.F. and T.G.) independently analyzed the transcripts using Atlas.ti (version 23.1.1, Atlas.ti Scientific Software Development GmbH; Berlin, Germany). First, open coding was performed after reading all interviews. Then, descriptive codes were formulated in barriers and facilitators and were recombined into the six domains (innovation, patient, healthcare professional, social setting, organization, and economic and financial) using axial coding. Finally, C.F. and T.G. discussed their findings until they achieved consensus. Disagreements were discussed with a third researcher (R.H.) until a consensus was reached.

### 2.3 Quantitative Phase

#### 2.3.1 Healthcare Professionals

Analogous to the focus groups, a panel of gynecologists, abdominal surgeons, urologists, and residents from these three specialties was recruited in the survey. The e‐survey was distributed through multiple channels using anonymous tokens between May 28, 2024, and August 23, 2024, so no identifiable information was collected. All members of the NVOG (Dutch Society of Obstetrics & Gynecology) (1154 gynecologists and 366 gynecology residents) were invited by email. Members of the NVvH (Dutch Society for Surgery) and the NVU (Dutch Society for Urology) were invited by a hyperlink included in the newsletter. Focus group participants were also eligible for the survey, as the quantitative analysis aimed to quantify identified barriers and facilitators rather than explore them in depth.

Approached healthcare professionals of the focus groups were asked to share the survey hyperlink with their colleagues. Additionally, the hyperlink was shared on LinkedIn and Twitter.

#### 2.3.2 Data Collection

An e‐Survey was developed (distributed by LimeSurvey Community Edition Version 6.5.4) by researchers and clinicians (C.F., T.G., and R.H.). This e‐Survey was based on the defined theoretical frameworks, the results of the focus groups with healthcare professionals, and the results of the (focus group)interviews with policymakers. The e‐Survey was subsequently pilot‐tested by two gynecologists, one surgeon, and one urologist and was approved by the multidisciplinary project team.

The e‐survey started with informed consent, followed by nine questions regarding the professional's characteristics and four on the knowledge about OC and OS. Depending on the selected specialty, participants received targeted questions. The questionnaire included 11 statements in the ‘Innovation’ subdomain, 1 in ‘Patient’, 27 in ‘Healthcare Professional’, 17 in ‘Organization’, 3 in ‘Social’, and 3 in ‘Economic and Political’. Answers were scored on a 4‐point Likert scale (1—strongly disagree to 4—strongly agree) or on a dichotomous agree/disagree scale. The “not applicable” option was omitted to ensure an answer. Answering all questions was obligatory, and survey completion took 10–15 min. Surveys were eligible for inclusion if at least one subsection beyond the professional's characteristics was completed.

#### 2.3.3 Data Analysis

**FIGURE 2:**
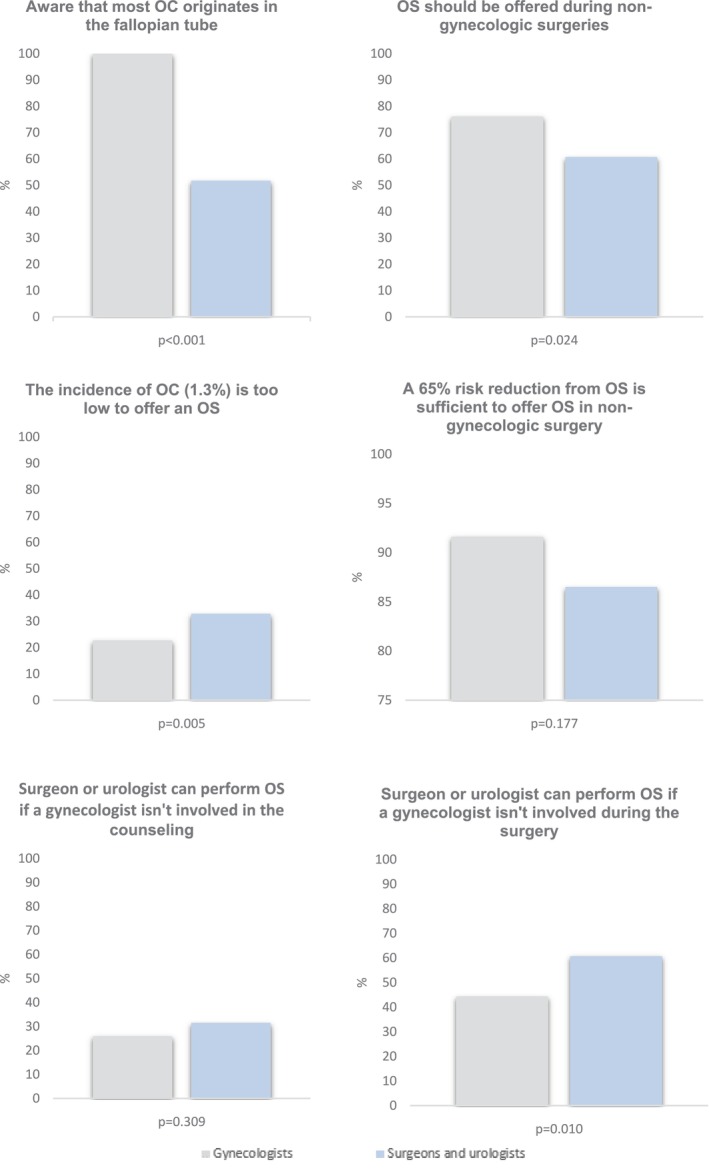
Opinion of healthcare professionals on opportunistic salpingectomy.

Data analysis was conducted using SPSS Statistics, version 29 (IBM Corp). Professional characteristics were analyzed descriptively and reported as percentages or mean values. Descriptive statistics were used to measure agreement with the statements. To ease interpretation, 4‐pointLikert scale was dichotomized (fully agree and agree; fully disagree and disagree) and the responses of abdominal surgeons and urologists were combined in Figure 2 and Table 1. For statistical analysis, chi‐square tests or exact tests (Fisher's exact or Fisher–Freeman–Halton Exact test) were performed.

**TABLE 1:** Professionals responses to statements regarding facilitators and barriers towards implementation of OS.

	Gynecologists^a^ (*n* = 220^b^)	Surgeons and urologists^a^ (*n* = 89^b^)
Innovation		
*Facilitators*		
The poor prognosis of OC	97.3%	93.3%
The lack of effective screening options for OC	94.7%	87.6%
The absence of effective treatment options for OC	94.2%	86.5%
The patient is already under anesthesia due to the initial surgery	85.3%	72.7%
The fallopian tube loses function after childbearing	87.6%	78.7%
OS does not affect the size surgery	—	47.2%
*Barriers*		
OS cannot be performed in all cases without additional risks	—	47.2%
OS cannot be performed without placements of extra trocars	—	41.6%
The eligible population for OS is unclear	—	58.4%
An emergency setting is not appropriate for an OS	80.9%	85.4%
Healthcare professional		
*Facilitators*		
I need uniform counseling material about OS	—	83.1%
I would like education on counseling of OS	—	74.2%
I would like education on how to technically perform an OS	—	74.2%
With the current evidence on the effectiveness of OS, I am prepared to implement it	96.4%	67.8%
*Barriers*		
I have insufficient surgery skills to perform OS	—	20.2%
I would experience time stress during consultation through additional counseling on OS	—	42.7%
I do not want to counsel about OS	—	39.3%
I have insufficient skills to counsel on OS	—	46.1%
I find it a problem when the gynecologist is not involved in counseling	—	43.8%
I have insufficient skills to counsel on reproductive desire	—	55.1%
I have insufficient skills to counsel on menopause	—	77.5%
I have insufficient skills to counsel on contraception	—	71.9%
I find it a problem when the gynecologist is not involved in the surgery	—	14.6%
I find it a problem when f I have to place one or more trocar(s) extra for an OS	—	28.1%
I do not want to perform OS because I would have less time for operations in my own field	—	25.8%
Organizational context		
*Barriers*		
I need more time for counseling on OS, which increases my consultation time	—	89.9%
I need more surgery time due to OS	—	92.1%
All fallopian tubes should be submitted for pathological analysis	—	66.3%
Social context		
*Facilitators*		
Consensus on OS within the departments would advance the implementation	99.5%	97.7%
Clear agreements on OS of urology and/or surgery with the gynecology department would advance the implementation	98.6%	98.8%
Incorporating OS into urological and surgical guidelines would advance the implementation	95.9%	89.7%
*Barriers*		
The capacity in healthcare is too limited to add an OS to it	17.3%	31.5%
I think waiting lists for regular surgery will increase by performing OS	12.3%	36.0%
Economic and political context		
*Barriers*		
I do not know how to invoice OS	57.7%	93.3%
I am only willing to perform an OS if it is proven cost‐effective	28.2%	64.4%
I will not perform an OS if I cannot invoice it	33.6%	64.4%

^a^ Including residents.

^b^ Less than 5% dropout during the questionnaire.

## 3 Results

### 3.1 Qualitative Phase

#### 3.1.1 Study Population: Healthcare Professionals

We approached 169 healthcare professionals (gynecologists, urologists, abdominal surgeons, and residents from aforementioned specialties) to participate in focus groups, of which 77 (45.6%) expressed interest in participating. In October 2023, four 60‐min focus group interviews were held, three with medical specialists and one with residents. Thirty‐eight professionals engaged in the sessions (participation rate 49.4%; 4–14 professionals per focus group). All participants completed the questionnaire (100.0%). The group (*n* = 38) consisted of 13 gynecologists (34.2%), 10 abdominal surgeons (26.3%), 6 urologists (15.8%), and 9 residents (3 in gynecology, 2 in surgery, and 4 in urology) (23.7%). Participants' are summarized in Table 2. The participants had a median work experience of 12 years and performed a median of 50 abdominal surgeries annually. Among healthcare professionals, 76.3% were aware of the fallopian tube's role in the origin of ovarian cancer, with this knowledge varying significantly by specialty: 100.0% of gynecologists, 50.0% of general surgeons, and 70.0% of urologists. Additionally, 52.6% had previously performed an OS, with 100.0% of gynecologists, 16.7% of surgeons, and 20.0% of urologists having done so.

**TABLE 2:** Baseline characteristics of healthcare professionals and residents in the focus groups and questionnaires.

Participating healthcare professionals	Focus group total (*N* = 38)	E‐survey total (*N* = 338)
Age^a^	41 (29–66)	45 (28–69)
Gender		
Male	22 (57.9%)	143 (42.3%)
Female	16 (42.1%)	195 (57.7%)
Specialty		
Gynecologist	13 (34.2%)	210 (62.1%)
Surgeon	10 (26.3%)	60 (17.8%)
Urologist	6 (15.8%)	28 (8.3%)
Resident	9 (23.7%)	40 (11.8%)
Gynecology	3 (7.9%)	25 (7.4%)
Surgery	2 (5.3%)	10 (3.0%)
Urology	4 (10.5%)	5 (1.5%)
Type of center working in		
University hospital	14 (36.8%)	70 (20.7%)
Non‐Academic teaching hospital	23 (60.5%)	190 (56.2%)
Non‐Academic non‐teaching hospital	3 (7.9%)	78 (23.1%)
Work experience in years^a^	12 (5–40)	17 (2–43)
No. of abdominal surgeries per year^a^	50 (0–350)	68 (0–600)
Knows about the role of the fallopian tube in the origin of ovarian cancer		
Total	29 (76.3%)	287 (84.9%)
Gynecologists^b^	16 (100%)	235 (100%)
Surgeons^b^	6 (50.0%)	39 (55.7%)
Urologists^b^	7 (70.0%)	13 (39.4%)
Previously performed opportunistic salpingectomy		
Total	20 (52.6%)	276 (81.7%)
Gynecologists^b^	16 (100%)	230 (97.9%)
Surgeons^b^	2 (16.7%)	33 (47.1%)
Urologists^b^	2 (20.0%)	13 (39.4%)

Abbreviation: *N*, number of participants.

^a^ Data are shown as medians.

^b^ Including residents.

### 3.2 Study Population: Policymakers

The focus group consisted of 11 participants, of which four were from medical professional associations (NVOG, NVvH and NVU) and seven were from patient associations (Radboudumc Patient Advisory Board for woman's cancer, Foundation Olijf, Foundation bekkenbodem 4all, patient association bladder‐and kidney cancer). In total, three individual interviews with different health insurance authorities (NZA, ZIN, insurance company VGZ) (*N* = 3) were conducted to provide a comprehensive perspective.

### 3.3 Barriers and Facilitators

**FIGURE 3:**
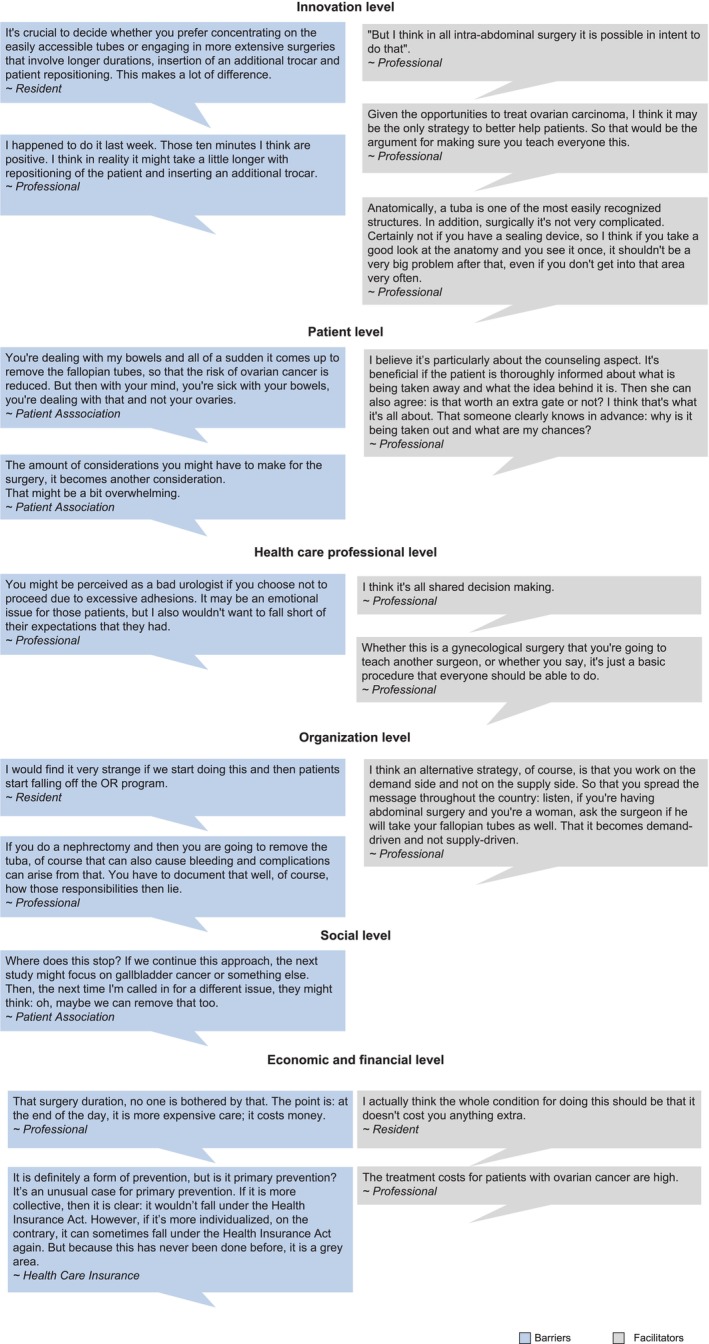
Illustrative quotations from professionals and policymakers concerning barriers and facilitators on implementation of OS in non‐gynecologic surgery.

In all domains, except for the social domain, healthcare professionals identified 38 barriers and 28 facilitators. Policymakers identified 37 barriers and 33 facilitators in all 6 domains (Table 3). Of these, 8 barriers and 7 facilitators were identified by both professionals and policymakers. Figure 3 provides illustrative quotations in implementing OS during non‐gynecologic surgeries by domain. Barriers that appeared consistently throughout all (focus group) interviews were: the increased workload due to the addition of OS, unclarity about invoicing for OS, and the variation in eligible women and types of surgeries, which hinders a uniform approach. Facilitators that were repeatedly mentioned included the poor prognosis of OC, the simplicity of the performance of OS, and the availability of counseling materials.

**TABLE 3:** Barriers and facilitators identified during interviews and focus groups by healthcare professionals and policymakers.

Domain	Barriers	Facilitators
Identified by professionals		
Innovation	Required contraception up until the surgery Increased complication risk due to additional operation area Increased importance of trocar positioning Possible need for additional trocar(s) Possible need to change the patients' position on the operating table Increased operating time Not suitable for the emergency setting Unclear if pathologic assessment of the tubes is necessary Unfeasibility of uniform strategy due to variety of eligible abdominal surgeries	Possible during all abdominal surgeries Simple procedure to perform Good overview of abdomen and tubes during OS Reduction of ovarian cancer risk Poor prognosis of ovarian cancer
Patient	Poor prognosis (as indication for initial surgery) Comorbidities Younger age Having to pay a personal fee	Postmenopausal status Shared decision making in counseling Explanation of rationale in counseling Availability of counseling materials (e.g., decision aid, videos, leaflets)
Healthcare professionals	Unaware of the possibility of OS Difference in effectiveness OS for different surgeries Lack of time to counsel for OS Forget to counsel for OS because it is not routine Unwillingness/lack of expertise to counsel about reproductive desire and menopause Unable to meet expectations of patients Unfamiliar with the execution of OS Insufficient skills to perform OS Responsibility for a surgery outside of their area of expertise Less time for their own surgeries Trained to operate conservatively Too much involvement of a gynecologist	Having knowledge about OS Information and education about OS Appropriate and clear selection criteria Request from patient to perform OS No complicated (dis)advantages to explain in counseling Availability of counseling materials (e.g., decision aid, videos, leaflets) Previous experience with surgeries in the gynecological area Many eligible women lead to a steep learning curve in performing OS Training on how to perform OS Support from the gynecological department
Social context	Variation in willingness among surgeons/urologists within and across hospitals	Academic setting in a hospital
Organizational context	Does not fit in current agreements on division of tasks of between medical specialties Unclarity about role of gynecologist Additional pressure on current capacity problems Less capacity for regular patients Needed additional surgical materials Increased surgical time Focus on production in surgical schedules Too little numbers per surgeon to become skilled in performing OS	Agreements on division of tasks of different medical specialties Minimized changes to current care and care paths Cooperation between gynecology and other involved specialties Involving nurses Additional time to perform OS
Economic and political context	Invoicing OS is unclear Higher costs due to increased surgical and counseling time Absence of cost‐effectiveness analysis of OS during non‐gynecologic surgery	
Identified by policymakers^a^		
Innovation (OS)	Low lifetime risk for ovarian cancer in the general population Possible need for additional trocar(s) Possible need to change the patients' position on the operating table Increased operating time Increased extensiveness of initial surgery Remaining residual risk for ovarian cancer after OS Unclear number needed to treat (NNT) in non‐gynecological surgeries Insufficient evidence of long‐term effects Limited scientific evidence within non‐gynecological surgeries	Simple procedure to perform Patient is already under anesthesia for initial surgery Reduction of ovarian cancer risk Poor prognosis of ovarian cancer Fallopian tubes has no function after completion of childbearing Early ovarian cancer detection currently not possible Limited treatment options for ovarian cancer High success rate of OS No short‐term disadvantages of OS No expected increased complication risk Limited increased extensiveness of initial surgery
Patient	Presents with a completely different complaint to the doctor Fear of earlier menopause Unwillingness to have healthy organs removed Lack of knowledge regarding the difference between ovaries and fallopian tubes Lack of knowledge regarding ovarian cancer Having to make too many decisions simultaneously Complicated choice	Family history of ovarian cancer Malignancy as indication for initial surgery Shared decision making in counseling Availability of counseling materials (e.g., decision aid, videos, leaflets) Confidence in treating physician High level of experience with OS of treating physician Counseling by their own physician Involvement of a gynecologist Sufficient time to consider the decision Easy choice
Health care professional	Insufficient knowledge about OS and ovarian cancer Insufficient skills to perform OS Experience of time pressure due to addition of OS Fear of increased complications	Appropriate and clear selection criteria Availability of counseling materials (e.g., decision aid, videos, leaflets) Performance of a clinical study to assess additional operation time and surgical complications
Organization	Increased surgical time Increased workload	Administration of OS Counseling by surgeon or urologist.
Social	Prevention programs must meet strict criteria Climate in healthcare is to act more conservative	National consensus on OS Communal policy about OS in departments and hospitals Inclusion of OS in the guidelines of the NVOG Inclusion of OS in surgical/urological guidelines
Economic and financial	Invoicing of OS is unclear Invoicing of OS is currently unfeasible Unclear which law covers OS invoicing Unclear how to make OS invoicing feasible Impossibility to open an additional invoice for OS next to initial invoice. Absence of a financing structure for additional preventive surgeries Similar surgery (sterilization) currently not covered by insurance Higher costs due to increased surgical and counseling time Higher cost due to Fallopian tube analysis by pathologists Absence of cost‐effectiveness analysis of OS during non‐gynecologic surgery	Individual with ovarian carcinoma incurs high treatment costs

Abbreviation: OS, opportunistic salpingectomy.

^a^ Policymakers: NVOG (Dutch society for Obstetrics and Gynecology), NVvH (Dutch society for Surgery), NVU (Dutch society for Urology), Foundation Olijf, Patient advisory board woman's cancer (Radboud UMC), Foundation bekkendbodem 4all, Patient association bladder and kidney cancer, NZA (Dutch Healthcare authority), ZIN (Dutch Healthcare Institute), Health insurance company.

### 3.4 Quantitative Phase

#### 3.4.1 Study Population: Healthcare Professionals

A total of 393 healthcare professionals started the questionnaire, of which 338 (86.0%) completed at least one subsection beyond professional's characteristics and were included in the analysis. This group comprised 235 gynecologists and gynecologic residents (69.5%), 70 abdominal surgeons and surgical residents (20.7%), and 33 urologists and urologic residents (9.8%), representing a diverse range of work experiences and hospital types (Table 2). A total of 306 healthcare professionals completed the entire questionnaire.

#### 3.4.2 Knowledge About OS

Knowledge about OS varied across the three types of specialties. Almost all professionals indicated they know the difference in function between the ovary and fallopian tube, and the loss of function of the fallopian tube after childbearing, 99.1% and 98.5%, respectively. The familiarity with the hypothesis that the majority of OC originates in the fallopian tube differed significantly (*p* < 0.001) across groups. While 100.0% of gynecologists were aware of the etiology of OC, only 51.7% of both surgeons and urologists were familiar with it. Similarly, 98.7% of gynecologists had previously performed an OS, compared to only 43.8% of surgeons and urologists (*p* < 0.001).

#### 3.4.3 Barriers and Facilitators in Six Domains

##### 3.4.3.1 Innovation (OS)

Of gynecologists, 76.0% agreed with the statement that an OS should be offered at all non‐gynecological surgeries, compared to 60.7% of surgeons and urologists (Figure 2). Facilitators identified in all specialties included the ovarian cancer risk reduction (90.2%), the fact that there are no effective screening methods (92.7%) or treatment options for OC (92.0%), as well as the fact that a patient is already under anesthesia for their initial surgery (81.8%) (Table 1). As barriers, the expectance of having to place extra trocars (41.6%), the expectation of more complications due to the addition of OS (47.2%), an emergency setting (82.2%) and an increased surgical time (92.1%) were identified.

##### 3.4.3.2 Patient

The eligibility of oncological patients for OS was considered a barrier by 19.7% of participants, who expressed concerns about offering OS to patients with a potentially limited life expectancy.

##### 3.4.3.3 Healthcare Professional

Identified barriers in this domain consist of 43.8% of both surgeons and urologists that consider the absence of a gynecologist during patient counseling to be problematic. Additionally, a majority of surgeons and urologists report feeling insufficiently skilled to counsel on topics such as reproductive desire, menopause, and contraception, 55.1%, 77.5%, and 71.9%, respectively. Of gynecologists, 25.8% agreed that a surgeon or urologist can perform an OS if a gynecologist is not involved in the counseling, compared to 31.5% of surgeons and urologists.

Facilitators include that 83.1% of surgeons and urologists expressed a need for uniform counseling material. During surgery, only 14.6% of urologists and surgeons consider the absence of a gynecologist problematic. However, 20.2% feel they lack the necessary skills to execute an OS, and 74.2% indicate a need for education on the technical execution of an OS. In contrast, only 44.4% of gynecologists agree that a surgeon or urologist can perform OS without a gynecologist being involved during the surgery. Based on the current available evidence on OS, 67.8% of urologists and surgeons are willing to perform it as an addition to the initial surgery.

##### 3.4.3.4 Organizational Context

An identified barrier is the need for additional time for counseling, which is expected by 89.9% of surgeons and urologists, of which 42.7% expect to experience extra time pressure when counseling for OS.

##### 3.4.3.5 Social Context

Barriers consist of an increased waiting list (19.2%) and a limited capacity to add OS in current healthcare (21.5%). A common policy across specialties (99.0%), clear agreements with the gynecology department about OS (98.6%) and including OS in surgical and urologic guidelines (94.1%) were identified as facilitators.

##### 3.4.3.6 Economic and Political

A significant barrier is the lack of clarity regarding the invoicing of OS, which affects 57.7% of gynecologists and 95.4% of surgeons and urologists. Other barriers include that 33.6% of gynecologists and 64.4% of surgeons and urologists are unwilling to perform an OS if they cannot bill for this, and the fact that 28.2% of gynecologists and 64.4% of surgeons and urologists will only perform an OS if this is proven to be cost effective.

## 4 Discussion

### 4.1 Findings

This mixed‐methods study indicates that the main barriers for implementation are the ambiguity regarding which populations/surgical types are eligible for OS, the perceived additional complication risks of OS, the increased workload as a result of adding OS, and the unclarity around invoicing an OS. Important facilitators are the poor prognosis of ovarian cancer (OC), the availability of uniform counseling materials, education on counseling and technical performance of OS, the involvement of a gynecologist during the counseling, and clear agreements between the departments within hospitals.

### 4.2 Interpretation

The implementation of OS in non‐gynecological surgery is a novel concept and a next step after the implementation of OS in gynecological surgeries. Research on the addition of OS to non‐gynecological surgeries is scarce [26].

From a professional perspective, a major barrier is the lack of clarity regarding the eligible populations for OS. Factors such as patient age, reproductive desire, menopausal status, and the number of previous surgeries that may increase the risk of adhesions could contribute to this ambiguity. Younger women may experience regret after a few years due to a renewed desire for children. Data among women who underwent sterilization at age 30 or younger express approximately twice as often regrets [27]. Therefore, we should remain cautious when offering OS to women under 30 years of age. Additionally, in patients with a history of abdominal adhesions or those with oncological conditions and reduced life expectancy, the question of whether the benefits of OS outweigh the risks is important. Incorporating clear eligibility criteria within surgical and urological guidelines tailored to the specific type of surgeries could enhance implementation, as stated by 94% of healthcare professionals.

Another identified barrier is that 43.8% of surgeons and urologists think the absence of a gynecologist during counseling is a significant issue, possibly due to feeling underprepared to discuss topics such as reproductive desire, menopause, and contraception, with 55%, 78%, and 72% acknowledging these gaps. However, a key facilitator is the need for uniform counseling materials and targeted education on OS. Therefore, providing education on OS by gynecologists could help bridge these knowledge gaps. Additionally, a decision aid could enhance counseling by addressing these topics comprehensively. Subsequently, an evaluation should be conducted to determine if counseling by a gynecologist is necessary, as this would involve an additional consultation, potentially increasing healthcare costs and causing longer waiting times for other patients.

Regarding the performance of the surgery itself, we identified several barriers in our questionnaire, such as the additional needed surgical time, increased complication risk, and having to reposition the patient and to insert extra trocars. A prospective study on OS in laparoscopic cholecystectomies in Austria showed that OS could be successfully performed in 93% of patients, and it took an extra 13 min to perform the additional procedure, without any OS‐related complications [14]. Additionally, a study in bariatric patients showed that in 81% of patients the fallopian tubes could be visualized, which took on average 3.5 min [28]. This could indicate that the expected increased complication risk and needed extra surgical time might be lower than expected by our participants.

In our questionnaire, 80% of surgeons and urologists indicate they think they have sufficient surgery skills to perform an OS. This corresponds with a study that showed that when an OS is performed independently by general surgeons, there are similar perioperative outcomes compared to the performance of OS with gynecological assistance [29]. In contrast, only 44% of gynecologists agree that a surgeon or urologist can perform OS without their involvement. Whether this reluctance stems from a lack of confidence in the counseling or surgical skills of surgeons or urologists, an unwillingness to share their area of expertise remains unclear, or concerns about potential financial losses remain unclear. A major facilitator identified by more than two‐thirds of surgeons and urologists is the need for education on the technical execution of OS. Therefore, successful implementation relies on effective collaboration between these specialties as well as appropriate education for surgeons and urologists. By optimizing collaboration and education, we will maximize success rates for a strategy in which OS can be performed exclusively by a surgeon or urologist, thereby streamlining logistics and potentially reducing costs.

One of the main concerns regarding the implementation of OS concerns invoicing and costs, which came up in all focus groups, interviews, and the questionnaire. Whereas only one third of gynecologists would not execute an OS when they cannot bill it, this proportion is about two thirds among surgeons and urologists, which makes this a relevant issue. Additionally, about two thirds of the surgeons and urologists will only perform OS when it is proven to be cost‐effective. Many studies have shown the cost‐effectiveness of OS in gynecological surgeries, but up until this point only two studies showed that the performance of OS during laparoscopic cholecystectomy is cost‐effective [15, 16]. For other eligible surgical indications, data is scarce [16, 26]. Therefore, cost‐effectiveness analyses should be conducted, incorporating a diverse range of operations and considering potential additional costs, such as extra consultations and the presence or absence of a gynecologist during the procedure. Based on these calculations, agreements should be established regarding the invoicing of OS.

### 4.3 Strengths and Weaknesses

This is the first study assessing barriers and facilitators for the implementation of an opportunistic salpingectomy during non‐gynecological surgeries. Results of this data are relevant for all healthcare professionals and policymakers who are willing to implement OS in non‐gynecological surgery, in which interest is increasing [26].

The main strength of our study is that we used mixed‐methods in order to qualify and quantify possible influencing factors from both healthcare professionals and policymakers. By using the results of our focus groups and interviews as a guide for the development of the e‐survey, we did not only identify barriers and facilitators, but also evaluated them on a larger scale to facilitate the establishment of an implementation strategy.

However, there are also limitations of this study. This research was performed in the Netherlands, where other regulations or opinions (e.g., regarding costs) could be applicable than in other countries. This might affect generalizability. However, we do feel that all barriers and facilitators are relevant in implementation in other countries as well, but the degree of applicability of some barriers or facilitators may vary across countries. Another limitation could be that surgeons and urologists did not have sufficient prior knowledge on OS; we tried to overcome this by providing all the necessary background information in the questionnaire needed to adequately answer all questions. Additionally, limited knowledge on OS outside of the gynecology specialty is common. Furthermore, a notable disadvantage of e‐surveys is the uncertainty regarding who exactly receives them, which prevents accurate calculation of the response rate. The response rate among surgeons and urologists appears significantly lower within this e‐survey, possibly because OS is less prominent within these specialties, which could lead to non‐response bias. To minimize this bias, we disseminated the e‐survey through multiple channels; however, response rates among these specialists remained lower. Lastly, the patient's perspective might be underexposed in this study, due to inclusion of healthcare professionals and patients' advocates. Therefore, this perspective is addressed in an ongoing in‐depth interview study being conducted with patients who have undergone non‐gynecological surgery.

To successfully implement OS in clinical practice, it is essential to address the identified barriers. Key steps include providing education to healthcare professionals and information for patients, developing appropriate counseling materials to support specialists, fostering collaboration between gynecologic and non‐gynecologic specialties, and achieving clarity on invoicing processes. Current efforts focus on developing tailored implementation strategies, such as adapting an existing decision aid for OS in the context of non‐gynecologic abdominal surgeries and creating informational materials, including a video for specialists outlining best practices for performing OS. Although invoicing challenges are context‐dependent and vary across countries, ongoing discussions with stakeholders aim to establish sustainable solutions within the healthcare system, ensuring equitable access to OS for all patients.

## 5 Conclusion

In this study, we identified barriers and facilitators relevant for the implementation of OS during non‐gynecological surgeries. Main barriers include the lack of knowledge, the unclarity of invoicing, and the increased workload, whereas main facilitators include the provision of education on counseling and the technical performance of OS and uniform counseling material.

## Author Contributions

**Charlotte Fisch:** conceptualization (lead), data curation (lead), formal analysis (lead), funding acquisition (equal), investigation (lead), methodology (lead), project administration (lead), writing – original draft (lead), writing – review and editing (lead). **Tamar Gootzen:** formal analysis (equal), investigation (equal), writing – original draft (equal), writing – review and editing (equal). **Joanne de Hullu:** conceptualization (equal), data curation (equal), funding acquisition (equal), investigation (equal), methodology (equal), supervision (equal), writing – review and editing (equal). **Philip de Reuver:** conceptualization (equal), funding acquisition (equal), methodology (equal), supervision (equal), writing – review and editing (equal). **Diederik Somford:** conceptualization (equal), methodology (equal), supervision (equal), writing – review and editing (equal). **Simon Nienhuijs:** conceptualization (equal), investigation (equal), writing – review and editing (equal). **Jurgen Piek:** conceptualization (equal), data curation (equal), funding acquisition (equal), investigation (equal), methodology (equal), supervision (equal), writing – review and editing (equal). **Rosella Hermens:** conceptualization (equal), data curation (equal), formal analysis (equal), funding acquisition (equal), investigation (equal), methodology (equal), supervision (equal), writing – review and editing (equal).

## Ethics Statement

This study was not subject to the Dutch ‘Medical Research Involving Human Subjects Act’, as assessed by the institutional review board of the Radboud University Medical Center (reference nr. 2023/16486).

## Conflicts of Interest

The authors declare no conflicts of interest.

## Supporting information


**Data S1.** COREQ (COnsolidated criteria for REporting Qualitative research) Checklist.

**Data S2.** CHERRIES (Checklist for Reporting Results of Internet E‐Surveys).

## Data Availability

The data that support the findings of this study are available from the corresponding author upon reasonable request.

## References

[1] L. A. Torre , B. Trabert , C. E. DeSantis , et al., “Ovarian Cancer Statistics, 2018,” CA: A Cancer Journal for Clinicians 68, no. 4 (2018): 284–296. 29809280 10.3322/caac.21456 PMC6621554

[2] Society AC , Key Statistics for Ovarian Cancer 2024, https://www.cancer.org/cancer/types/ovarian‐cancer/about/key‐statistics.html.

[3] S. Lheureux , C. Gourley , I. Vergote , and A. M. Oza , “Epithelial Ovarian Cancer,” Lancet 393, no. 10177 (2019): 1240–1253. 30910306 10.1016/S0140-6736(18)32552-2

[4] I. J. Jacobs , U. Menon , A. Ryan , et al., “Ovarian Cancer Screening and Mortality in the UK Collaborative Trial of Ovarian Cancer Screening (UKCTOCS): A Randomised Controlled Trial,” Lancet 387, no. 10022 (2016): 945–956. 26707054 10.1016/S0140-6736(15)01224-6 PMC4779792

[5] J. M. Piek , R. H. Verheijen , P. Kenemans , L. F. Massuger , H. Bulten , and P. J. van Diest , “BRCA1/2‐Related Ovarian Cancers Are of Tubal Origin: A Hypothesis,” Gynecologic Oncology 90, no. 2 (2003): 491. 10.1016/s0090-8258(03)00365-2 12893227

[6] R. J. Kurman and I. M. Shih , “Molecular Pathogenesis and Extraovarian Origin of Epithelial Ovarian Cancer—Shifting the Paradigm,” Human Pathology 42, no. 7 (2011): 918–931. 21683865 10.1016/j.humpath.2011.03.003 PMC3148026

[7] J. M. Piek , P. J. van Diest , R. P. Zweemer , et al., “Dysplastic Changes in Prophylactically Removed Fallopian Tubes of Women Predisposed to Developing Ovarian Cancer,” Journal of Pathology: A Journal of the Pathological Society of Great Britain and Ireland 195, no. 4 (2001): 451–456. 10.1002/path.1000 11745677

[8] G. E. Hanley , C. L. Pearce , A. Talhouk , et al., “Outcomes From Opportunistic Salpingectomy for Ovarian Cancer Prevention,” JAMA Network Open 5, no. 2 (2022): e2147343‐e. 35138400 10.1001/jamanetworkopen.2021.47343 PMC8829665

[9] H. Falconer , L. Yin , H. Gronberg , and D. Altman , “Ovarian Cancer Risk After Salpingectomy: A Nationwide Population‐Based Study,” International Journal of Gynecological Cancer 24, no. 9 (2014): 1581–1581. 10.1093/jnci/dju410 25628372

[10] C. Madsen , L. Baandrup , C. Dehlendorff , and S. K. Kjær , “Tubal Ligation and Salpingectomy and the Risk of Epithelial Ovarian Cancer and Borderline Ovarian Tumors: A Nationwide Case–Control Study,” Acta Obstetricia et Gynecologica Scandinavica 94, no. 1 (2015): 86–94. 25256594 10.1111/aogs.12516

[11] S. E. Dilley , J. M. Straughn, Jr. , and C. A. Leath, III , “The Evolution of and Evidence for Opportunistic Salpingectomy,” Obstetrics & Gynecology 130, no. 4 (2017): 814–824. 28885426 10.1097/AOG.0000000000002243

[12] A. Ntoumanoglou‐Schuiki , G. Tomasch , R. Laky , N. Taumberger , V. Bjelic‐Radisic , and K. Tamussino , “Opportunistic Prophylactic Salpingectomy for Prevention of Ovarian Cancer: What Do National Societies Advise?,” European Journal of Obstetrics & Gynecology and Reproductive Biology 225 (2018): 110–112. 29704813 10.1016/j.ejogrb.2018.03.043

[13] J. W. Nunoo‐Mensah , M. Rosen , L. S. Chan , N. Wasserberg , and R. W. Beart , “Prevalence of Intra‐Abdominal Surgery: What Is an Individual's Lifetime Risk?,” Southern Medical Journal 102, no. 1 (2009): 25–29. 19077782 10.1097/SMJ.0b013e318182575b

[14] G. Tomasch , M. Lemmerer , S. Oswald , et al., “Prophylactic Salpingectomy for Prevention of Ovarian Cancer at the Time of Elective Laparoscopic Cholecystectomy,” British Journal of Surgery 107, no. 5 (2020): 519–524. 32129898 10.1002/bjs.11419 PMC7154767

[15] K. Matsuo , L. Chen , S. Matsuzaki , et al., “Opportunistic Salpingectomy at the Time of Laparoscopic Cholecystectomy for Ovarian Cancer Prevention: A Cost‐Effectiveness Analysis,” Annals of Surgery 277, no. 5 (2023): E1116–E1123. 35129467 10.1097/SLA.0000000000005374

[16] B. N. Hughes , T. J. Herzog , J. Brown , and R. W. Naumann , “Opportunistic Salpingectomy at Time of Nongynecologic Elective Procedures Could Reduce Ovarian Cancer–Related Costs and Mortality,” Journal of Gynecologic Surgery 38, no. 1 (2022): 43–48.

[17] M. Rius , J. Carugno , M. S. Abrao , and F. Carmona , “Opportunistic Salpingectomy for Ovarian Cancer Prevention: A Call for Action,” Journal of the American College of Surgeons 237, no. 2 (2023): 376–378. 37042549 10.1097/XCS.0000000000000713

[18] R. M. Kahn , S. Gordhandas , K. Godwin , et al., “Salpingectomy for the Primary Prevention of Ovarian Cancer: A Systematic Review,” JAMA Surgery 158, no. 11 (2023): 1204–1211. 37672283 10.1001/jamasurg.2023.4164 PMC11185162

[19] R. Grol and J. Grimshaw , “Evidence‐Based Implementation of Evidence‐Based Medicine,” Joint Commission Journal on Quality Improvement 25, no. 10 (1999): 503–513. 10522231 10.1016/s1070-3241(16)30464-3

[20] R. Grol and M. Wensing , “What Drives Change? Barriers to and Incentives for Achieving Evidence‐Based Practice,” Medical Journal of Australia 180 (2004): S57–S60. 15012583 10.5694/j.1326-5377.2004.tb05948.x

[21] S. A. Flottorp , A. D. Oxman , J. Krause , et al., “A Checklist for Identifying Determinants of Practice: A Systematic Review and Synthesis of Frameworks and Taxonomies of Factors That Prevent or Enable Improvements in Healthcare Professional Practice,” Implementation Science 8 (2013): 1–11. 23522377 10.1186/1748-5908-8-35 PMC3617095

[22] A. Tong , P. Sainsbury , and J. Craig , “Consolidated Criteria for Reporting Qualitative Research (COREQ): A 32‐Item Checklist for Interviews and Focus Groups,” International Journal for Quality in Health Care 19, no. 6 (2007): 349–357. 17872937 10.1093/intqhc/mzm042

[23] G. Eysenbach , “Improving the Quality of Web Surveys: The Checklist for Reporting Results of Internet E‐Surveys (CHERRIES),” Journal of Medical Internet Research 6 (2004): e34. 15471760 10.2196/jmir.6.3.e34 PMC1550605

[24] R. E. Stalmeijer , N. McNaughton , and W. N. van Mook , “Using Focus Groups in Medical Education Research: AMEE Guide No. 91,” Medical Teacher 36, no. 11 (2014): 923–939. 25072306 10.3109/0142159X.2014.917165

[25] Leven Vig , Eerste Hulp Bij … FOCUSGROEPEN2018 2023, https://www.gezondleven.be/files/gezondheidsbevordering/Gezond‐Leven‐2018_‐Eerste‐Hulp‐Bij‐__‐focusgroepen.pdf.

[26] K. Verhoeff , K. Sorouri , J. Y. Kung , S. Pin , and M. Strickland , “Opportunistic Salpingectomy at the Time of General Surgery Procedures: A Systematic Review and Narrative Synthesis of Current Knowledge,” Surgeries 5, no. 2 (2024): 248–263.

[27] K. M. Curtis , A. P. Mohllajee , and H. B. Peterson , “Regret Following Female Sterilization at a Young Age: A Systematic Review,” Contraception 73, no. 2 (2006): 205–210. 16413851 10.1016/j.contraception.2005.08.006

[28] H. Sagmeister , D. Pucher , S. Oswald , F. Tadler , J. Strutzmann , and K. Tamussino , “Might Prophylactic Salpingectomy Be Possible During Bariatric Surgery? (“Can We See the Tubes?”),” Geburtshilfe und Frauenheilkunde 83, no. 4 (2023): 19.

[29] E. Myriokefalitaki , E. L. Moss , M. Thomas , and Q. Davies , “Should We Be Offering Women Undergoing Elective Colorectal Cancer Surgery a Prophylactic Salpingo‐Oophorectomy?,” International Journal of Gynecological Cancer 24, no. 9 (2014): 352–352.24407575

