# Supplementary material for: Opportunistic Salpingectomy in Non‐Gynecologic Surgeries: Barriers and Facilitators From a Healthcare Provider Perspective
Source: Cancer Med. 2025 May 9;14(9):e70945. doi: 10.1002/cam4.70945 (PMC12875002; doi:10.1002/cam4.70945)
Supplement: Supplementary file 2 — Data S2. CHERRIES (Checklist for Reporting Results of Internet E‐Surveys). [file CAM4-14-e70945-s002.docx]

**Checklist for Reporting Results of Internet E-Surveys (CHERRIES)**

| ***Checklist Item*** | ***Explanation*** | ***Page Number*** |
| --- | --- | --- |
| Describe survey design | Nationwide cross-sectional survey among all specialist and residents in surgery (gynecologists, general surgeons, urologists). | 9 |
| IRB approval | IRB approval was not applicable for this study. | 6 |
| Informed consent | The participants were invited to complete the survey by email. The email contained information about the content of the survey, including aim, and anonymous data handling. | 9 |
| Data protection | For data collection the web-based tool LimeSurvey was used. All data was automatically converted to anonymous tokens, therefore it was impossible to link e-mail addresses to the corresponding surveys. In this way total anonymity of participants was secured. | 9 |
| Development and testing | The survey was developed by a team of researchers and clinicians based on the interviews and current literature, subsequently it was extensively pilot-tested to assess the usability and technical functionality. | 9 |
| Open survey versus closed survey | Open survey | 9 |
| Contact mode | The (potential) participants were contacted by e-mail, newsletter, Twitter or LinkedIn. | 9 |
| Web/E-mail | The survey was web-based (LimeSurvey) and data was automatically transferred to a database. | 9 |
| Context | Not applicable. | - |
| Mandatory/voluntary | It was a voluntary survey. | 9 |
| Incentives | No incentives were offered. | - |
| Time/Date | Participants were invited to complete the survey between May and August 2024 | 9 |
| Randomization of items or questionnaires | Not applicable. |  |
| Adaptive questioning | The survey questions were apapted depending on the selected specialty (gynecology, surgery or urology). | 9 |
| Number of Items | The survey contained a total of 74 questions | 9 |
| Number of screens (pages) | The questionnaire was distributed across seven pages (screens). | - |
| Completeness check | The questions in the survey were all mandatory to complete before entering the next page (screen) with questions (an automatic option in LimeSurvey) | 10 |
| Review step | The questions in the survey were all mandatory to complete before entering the next page (screen) with questions (an automatic option in LimeSurvey) | - |
| Unique site visitor | Not applicable | - |
| View rate (Ratio of unique survey visitors/unique site visitors) | Not applicable | - |
| Participation rate (Ratio of unique visitors who agreed to participate/unique first survey page visitors) | Not applicable | - |
| Completion rate (Ratio of users who finished the survey/users who agreed to participate) | The completion rate was 78% | - |
| Cookies used | Not applicable because of the unique tokens | - |
| IP check | Not applicable | - |
| Log file analysis | Multiple entries were not allowed. | - |
| Registration | Not applicable. | - |
| Handling of incomplete questionnaires | Surveys were eligible for inclusion if at least one subsection (besides the baseline characteristics) was completed. | 10 |
| Questionnaires submitted with an atypical timestamp | Not applicable. | - |
| Statistical correction | Not applicable. | - |

This checklist has been modified from Eysenbach G. Improving the quality of Web surveys: the Checklist for Reporting Results of Internet E-Surveys (CHERRIES). J Med Internet Res. 2004 Sep 29;6(3):e34 [erratum in J Med Internet Res. 2012; 14(1): e8.]. Article available at [https://www.jmir.org/2004/3/e34](https://www.jmir.org/2004/3/e34/)/; erratum available <https://www.jmir.org/2012/1/e8/>. Copyright ©Gunther Eysenbach. Originally published in the [Journal of Medical Internet](http://www.jmir.org/) Research, 29.9.2004 and 04.01.2012.
